# Development and validation of a pyroptosis-related genes signature for risk stratification in gliomas

**DOI:** 10.3389/fgene.2023.1087563

**Published:** 2023-02-13

**Authors:** Penggang Sun, Xinyu Wang, Junzhe Zhong, Daohan Yu, Hanwen Xuan, Tianye Xu, Dan Song, Changxiao Yang, Pandeng Wang, Yuxiang Liu, Xiangqi Meng, Jinquan Cai

**Affiliations:** Department of Neurosurgery, The Second Affiliated Hospital of Harbin Medical University, Harbin, China

**Keywords:** glioma, pyroptosis, gene signature, overall survival, immune status

## Abstract

**Background:** Glioma is a highly heterogeneous disease, causing the prognostic prediction a challenge. Pyroptosis, a programmed cell death mediated by gasdermin (GSDM), is characterized by cell swelling and the release of inflammatory factors. Pyroptosis occurs in several types of tumor cells, including gliomas. However, the value of pyroptosis-related genes (PRGs) in the prognosis of glioma remains to be further clarified.

**Methods:** In this study, mRNA expression profiles and clinical data of glioma patients were acquired from TCGA and CGGA databases, and one hundred and eighteen PRGs were obtained from the Molecular Signatures Database and GeneCards. Then, consensus clustering analysis was performed to cluster glioma patients. The least absolute shrinkage and selection operator (LASSO) Cox regression model was used to establish a polygenic signature. Functional verification of the pyroptosis-related gene GSDMD was achieved by gene knockdown and western blotting. Moreover, the immune infiltration status between two different risk groups were analyzed through the “gsva” R package.

**Results:** Our results demonstrated that the majority of PRGs (82.2%) were differentially expressed between lower-grade gliomas (LGG) and glioblastoma (GBM) in the TCGA cohort. In univariate Cox regression analysis, eighty-three PRGs were shown to be associated with overall survival (OS). A five-gene signature was constructed to divide patients into two risk groups. Compared with patients in the low-risk group, patients in the high-risk group had obviously shorter OS (*p* < 0.001). Also, we found that the high-risk group showed a higher infiltrating score of immune cells and immune-related functions. Risk score was an independent predictor of OS (HR > 1, *p* < 0.001). Furthermore, knockdown of GSDMD decreased the expression of IL-1β and cleaved caspase-1.

**Conclusion:** Our study constructed a new PRGs signature, which can be used to predict the prognosis of glioma patients. Targeting pyroptosis might serve as a potential therapeutic strategy for glioma.

## 1 Introduction

Glioma, the most common primary central nervous system (CNS) malignancy, is characterized by extreme heterogeneity, short survival and high recurrence rate (Louis et al., 2016; Zhang et al., 2021). GBM, the most predominant pathological type of glioma, is highly malignant and aggressive, with a median patient survival of only 12–14 months and a 5-year survival rate of less than 10% (Jiang et al., 2016). The main reasons for the poor prognosis of patients include strong tumor cell proliferation and invasion ability, temozolomide chemotherapy resistance, and tumor microenvironment immunosuppression (Heimberger et al., 2008; Perazzoli et al., 2015; Cai et al., 2018; Luoto et al., 2018; Chen et al., 2019). According to statistics, in 2016, there were 330,000 cases of CNS tumors and 227,000 deaths worldwide (GBD, 2016 Brain and Other CNS Cancer Collaborators, 2019). In recent years, research developments on the progression and treatment of gliomas have continued to emerge (Qin et al., 2021; Sun et al., 2021; Zhao et al., 2022). Currently, the clinical treatment strategy for glioma patients is mainly surgical resection, supplemented by concurrent radiotherapy and chemotherapy (Stupp et al., 2008; Jiang et al., 2016; Wang et al., 2020a). Additionally, new treatment methods including molecular targeted therapy and immunotherapy gradually emerged (Wu et al., 2019; Li et al., 2020a; Meng et al., 2020; Wu et al., 2020). Molecular markers also play an important role in the diagnosis and treatment of gliomas, which can not only serve as targets for drug therapy, but also guide surgical treatment (Li et al., 2020b). However, overcoming the susceptibility for relapse and poor prognosis of glioma patients remains a challenge. The high heterogeneity of gliomas and the limitation of the diagnostic modalities also create challenges for the prognostic evaluation of patients. Consequently, it is necessary to further explore effective prognostic evaluation methods and promising therapeutic targets.

Pyroptosis is a kind of programmed cell death induced by caspases (Vande and Lamkanfi, 2016), which causes cell swelling, cell membrane rupture and intracellular release of proinflammatory substances (Fink and Cookson, 2007). Unlike apoptosis, pyroptosis requires the involvement of the GSDM family as executioners to mediate cell swelling (Feng et al., 2018). With the discovery of the GSDM family, the scope of research on pyroptosis has continued to expand. Pyroptosis can be activated through the following two main approaches: GSDMD-dependent pyroptosis regulated by caspase-1/4/5/11 and GSDME-dependent pyroptosis regulated by caspase-3 (Shi et al., 2015; Liu and Lieberman, 2017; Wang et al., 2017; Xu et al., 2018). Pyroptosis is closely related to a variety of human diseases, especially malignancies. The relationship between pyroptosis and tumors varies with different tissues and genetic backgrounds (Yu et al., 2021). For example, in esophageal squamous cell carcinoma, metformin can activate the pyroptosis process through the mir-497/proline, glutamate and leucine protein-1 pathway (Wang et al., 2019). In addition, lncRNA *RP1-85F18.6* is highly expressed in colorectal cancer tissues and can inhibit colorectal cancer cell pyroptosis (Ma et al., 2018). In gliomas, knockdown of hsa_circ_0001836 significantly increased the expression of NLRP1, cleaved caspase-1 and GSDMD-N, and induced the pyroptosis of glioma cells (Liu et al., 2021). Besides, miRNA-214 can inhibit the proliferation and migration of glioma cells by targeting caspase-1, which is involved in pyroptosis (Jiang et al., 2017). These studies showed that pyroptosis appeared in a wide variety of tumors, including gliomas. However, distinct roles of pyroptosis and PRGs in glioma remain poorly studied, and whether they are related to the prognosis of patients with glioma needs further verification.

In this study, the mRNA expression profiles and detailed clinical data of glioma patients were obtained from public databases (TCGA and CGGA). Subsequently, we performed differentially expressed gene analysis and univariate Cox regression analysis to excavate fifty-eight PRGs. Finally, a five-gene signature was constructed through LASSO regression analysis. We further validated the function of GSDMD, one of the five marker genes, in pyroptosis using gene knockdown and western blotting. Additionally, the single-sample gene set enrichment analysis (ssGSEA) was used to explore immune infiltration status of different risk groups. Our results indicated that PRGs play a crucial biological role in glioma and therefore may be promising prognostic biomarkers and targets for glioma.

## 2 Materials and methods

### 2.1 Data collection

RNA sequencing and clinical data were obtained by TCGA combined LGG/GBM dataset (*n* = 467 for LGG and 168 for GBM) retrieved from the UCSC Xena Browser, which is used as a training cohort. Meanwhile, RNA-seq transcriptome data and clinical characteristics of mRNAseq_325, mRNAseq_693 dataset (*n* = 630 for LGG and 388 for GBM) were obtained from CGGA (http://www.cgga.org.cn), which is used as a validation cohort.

Then, one hundred and eighteen genes involved in pyroptosis-related gene sets were obtained and showed in Supplementary Table S1, which included gene sets from GSEA on the Molecular Signatures Database (GOBP_PYROPTOSIS, REACTOME_PYROPTOSIS, http://www.broadinstitute.org/gsea/msigdb/index.jsp), and genes in GeneCards with relevance scores exceeded than 1.0. (https://www.genecards.org/).

### 2.2 Consensus clustering based on PRGs

According to the PRGs expression, glioma patients were clustered through the ConsensusClusterPlus R package. The number of clusters ranges from 2 to 9. We used cumulative-distribution function (CDF), delta area and consensus matrix to determine the optimal number of subtypes. Then, Kaplan-Meier method was applied to compare OS between glioma subtypes.

### 2.3 Construction and validation of a PRGs signature

Samples with complete survival information of TCGA (*n* = 692) and CGGA (*n* = 929) were considered to perform the univariate Cox analysis, the false discovery rate (FDR) < 0.05 was used to identify genes that associated with survival. Subsequently, the FDR <0.05 was applied to recognize differentially expressed genes (DEGs) between LGG and GBM. Then, we constructed protein-protein interaction (PPI) network for the prognostic-related DEGs using STRING and Cytoscape software (version 3.9.0). Furthermore, Cytoscape’s Cytohubba plug-in combined with Maximal Clique Centrality (MCC) method is used to identify hub nodes. The LASSO L1-penalized Cox regression method was used for variable selection by setting the one thousand simulations in “glmnet” package of R (Tibshirani, 1997; Friedman et al., 2010; Zhou et al., 2019). The risk score, based on the gene expression scores and corresponding regression coefficients, was calculated by the following formula: Risk score = ∑*ni* = ∑*Coefi × xi*, where *xi* represents the normalized expression level of target gene *i* and *Coefi* refers to corresponding regression coefficient. According to the median value of the risk score, all patients were further divided into high-risk or low-risk groups. For dimensionality reduction and data visualization, principal component analysis (PCA) was performed using the ‘prcomp’ function in the STATS package and t-distributed Stochastic Neighbour Embedding (t-SNE) analysis was applied using the Rtsne package, separately.

### 2.4 ssGSEA functional analysis

To explore the immune infiltration status related to the low-risk and high-risk groups, the ssGSEA in the “gsva” R package was implemented to calculate the infiltration score of sixteen immune cell types and the activity of thirteen immune-related functions (Subramanian et al., 2005; Farshadpour et al., 2012; Vacchelli et al., 2014; Meadows and Zhang, 2015; Vigneron et al., 2015; Hugo et al., 2016). Besides, we analyzed the correlation between the expressions of signature genes and immune infiltrating cells through ssGSEA.

### 2.5 Cell culture and transfection

Human glioma cells (U87 and LN229) were purchased from the Chinese Academy of Sciences Cell Bank (Shanghai, China). Cell lines were cultured in Dulbecco’s modified Eagle’s medium (DMEM) or DMEM/F12 with 10% fetal bovine serum (Gibco, United States) under a humidified atmosphere of 5% CO2 at 37°C. These cells were transfected with siRNAs by using ribo*FECT* ^TM^ CP (RiboBio, Guangzhou, China). Specifically, 5 × 10^5^ cells were seeded in 6-well plates overnight and transfected with siRNA targeting GSDMD (RiboBio, Guangzhou, China). Validation of siRNA was detected by western blotting.

### 2.6 Western blot

Glioma cells were lysed using RIPA buffer (Solarbio) with protease inhibitors, then centrifuged at 13,000 rpm for 30 min at 4°C. Concentrations of total protein were measured with the spectrophotometer (NanoDrop). Protein samples were subjected to sodium dodecyl sulfate polyacrylamide gel (EpiZyme Scientific) electrophoresis and transferred onto PVDF membranes. The PVDF membranes were blocked in a 5% milk-TBST solution and incubated overnight at 4°C with primary antibodies. After incubation with HRP-labeled secondary antibodies at room temperature for 1 h, the protein bands were visualized using a ChemiDocTM MP Imaging System (BioRad).

### 2.7 Statistical analysis

The *t*-test was utilized to compare gene expression between samples from LGG and GBM. The ssGSEA scores of immune infiltrating cells and immune-related functions between the two risk groups were compared by Mann-Whitney test, and Benjamini & Hochberg method was used to adjust *p*-value. Kaplan-Meier analysis was used to compare OS between high-risk and low-risk groups. Univariate and multivariate Cox regression analyses were used to determine independent predictors of OS. Data analysis of this study was performed by R software (version 4.0.5).

## 3 Results

**FIGURE 1:**
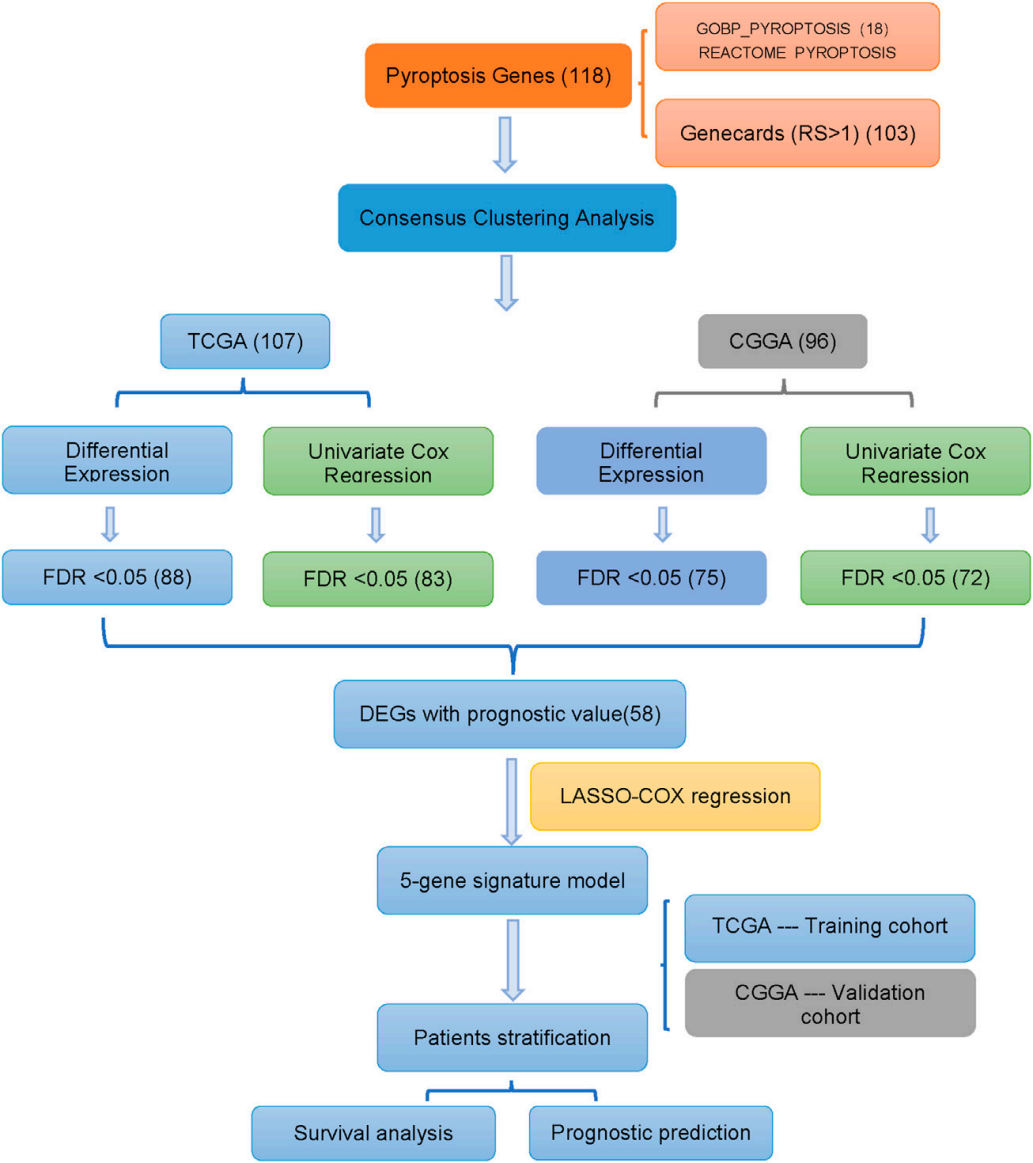
Flow chart of data collection and analysis.

Figure 1 shows the flow chart of our work. The study involved 692 glioma patients in the TCGA cohort and 929 glioma patients in the CGGA cohort. Table 1 summarizes the clinical characteristics of these patients.

**TABLE 1:** The clinical characteristics of glioma patients.

Characteristic	Levels	TCGA	CGGA
Total number, N		696	1018
WHO grade, n (%)	G2	224 (35.3%)	291 (28.6%)
	G3	243 (38.3%)	334 (32.8%)
	G4	168 (26.5%)	388 (38.1%)
IDH status, n (%)	Wild-type	246 (35.9%)	435 (42.7%)
	Mutant	440 (64.1%)	531 (52.2%)
1p/19q, n (%)	Codeletion	171 (24.8%)	213 (20.9%)
	Non-codeletion	518 (75.2%)	728 (71.5%)
Gender, n (%)	Female	298 (42.8%)	417 (41.0%)
	Male	398 (57.2%)	601 (59.0%)
Age, n (%)	≤60	553 (79.5%)	922 (90.6%)
	>60	143 (20.5%)	95 (9.3%)
Histological type, n (%)	Astrocytoma	195 (28%)	175 (17.2%)
	Glioblastoma	168 (24.1%)	388 (38.1%)
	Oligoastrocytoma	134 (19.3%)	9 (0.9%)
	Oligodendroglioma	199 (28.6%)	112 (11.0%)
	Anaplastic oligodendro		94 (9.2%)
	Anaplastic astrocytoma		214 (21.0%)
	Anaplastic oligoastrocytoma		21 (2.1%)
OS event, n (%)	Alive	424 (60.9%)	398 (39.1%)
	Dead	272 (39.1%)	539 (52.9)
DSS event, n (%)	Alive	431 (63.9%)	NA
	Dead	244 (36.1%)	NA
PFI event, n (%)	Alive	350 (50.3%)	NA
	Dead	346 (49.7%)	NA
Age, median (IQR)		45 (34, 59)	42 (35, 51)

### 3.1 Glioma subtypes based on consensus clustering analysis

**FIGURE 2:**
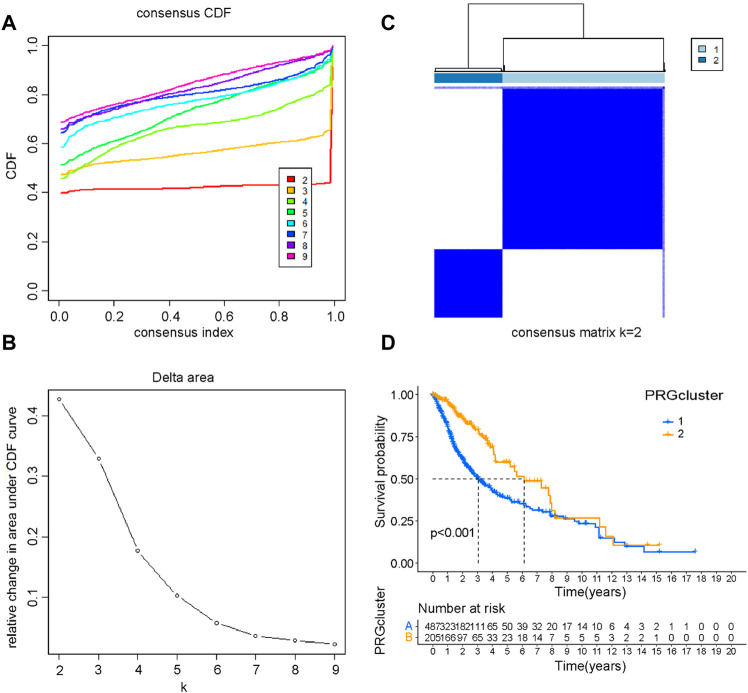
Consensus clustering of PRGs. **(A)** The empirical CDF plot with *k* = 2–9. **(B)** The CDF AUC with *k* = 2–9. **(C)** Consensus clustering matrix with *k* = 2. **(D)** The Kaplan-Meier survival curve of glioma patients in the cluster 1 and cluster 2.

To explore the prognostic implications of PRGs, we performed consensus clustering analysis with glioma patients in the training cohort. When the clustering variable (*k*) equaled 2, the empirical CDF plot revealed the lowest rangeability in the consensus index range of 0.1–0.9 and the delta area scored highest (Figures 2A, B). Also with *k* = 2, the consensus matrix plot showed the highest consistency (Figure 2C). Therefore, glioma patients were divided into two subtypes, namely, cluster 1 and cluster 2. Interestingly, patients in cluster 2 group had a better OS than that in cluster 1 group (Figure 2D). The clinical characteristics of two clusters were shown in Supplementary Table S2. Detailly, a total of 625 glioma patients were classified into cluster 1 and cluster 2 groups. The differences in WHO grade, IDH status and age between the two glioma subtypes were statistically significant. In contrast, there were no significant differences in 1p/19q and gender between the two subgroups.

### 3.2 Identification of prognostic DEGs in the TCGA and the CGGA cohort

**FIGURE 3:**
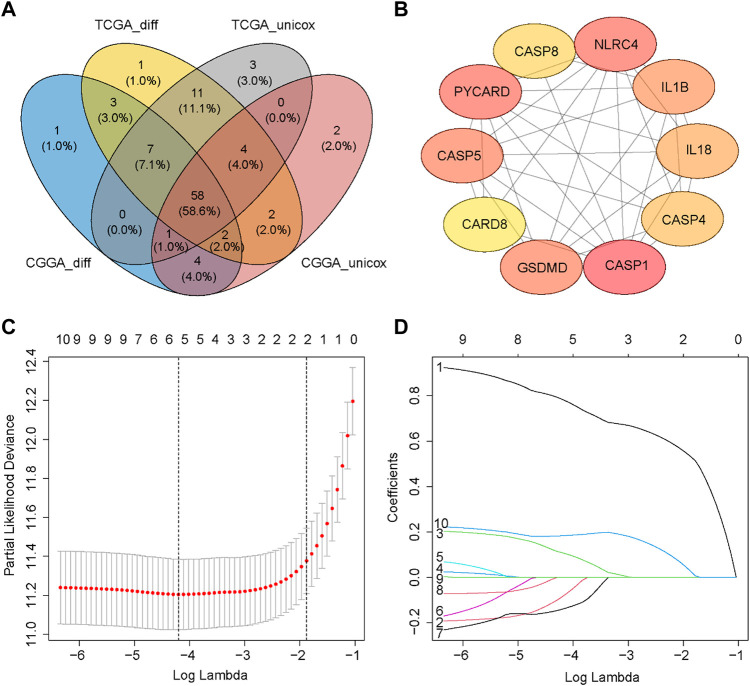
Identification of prognostic DEGs and construction of the PRGs signature. **(A)** Wayne Diagram shows the result of the intersection of four gene sets. **(B)** PPI network of the top 10 hub nodes. **(C)** Selection of the penalty parameter (*λ*) in the LASSO model *via* ten-foldcross-validation. The dotted vertical lines are plotted at the optimal values following the minimum criteria (left) and “one standard error” criteria (right). **(D)** LASSO coefficient profiles of the expression of ten candidate genes.

Most of the PRGs (88/107, 82.2%) showed significantly differential expression between LGG and GBM in the TCGA cohort. In univariate Cox regression analysis, eighty-three of these genes (83/107, 77.6%) were associated with OS. Similarly, we analyzed DEGs (75/96, 78.1%) and performed univariate Cox regression analysis (72/96, 75.0%) in the CGGA cohort. A total of fifty-eight PRGs were acquired by taking the intersection of four gene sets from two cohorts (Figure 3A). Then, we obtained the interaction information of forty-eight proteins from STRING with the comprehensive score ≥0.7 as the screening condition and constructed a PPI network through Cytoscape. Next, the top 10 hub nodes were identified using the Cytoscape’s Cytohubba plug-in (Figure 3B). The information of top 10 prognostic DEGs was presented in Table 2.

**TABLE 2:** The information of top 10 prognostic DEGs.

Gene Id	TCGA				CGGA			
	HR	HR.95L	HR.95H	P-value	HR	HR.95L	HR.95H	P-value
CASP8	3.187	2.724	3.730	2.33E-47	1.887	1.716	2.076	3.45E-39
CASP4	2.525	2.264	2.816	4.13E-62	1.504	1.411	1.604	1.76E-35
CASP5	2.501	2.135	2.931	7.96E-30	2.212	1.871	2.615	1.50E-20
GSDMD	2.315	2.071	2.587	2.14E-49	1.211	1.142	1.284	1.42E-10
CASP1	2.078	1.866	2.315	2.04E-40	1.451	1.345	1.566	1.09E-21
NLRC4	2.026	1.724	2.382	1.03E-17	1.674	1.444	1.940	8.67E-12
CARD8	1.984	1.597	2.465	6.04E-10	1.826	1.629	2.048	7.46E-25
PYCARD	1.890	1.672	2.135	1.79E-24	1.169	1.100	1.242	4.24E-07
IL18	1.739	1.553	1.947	7.52E-22	1.227	1.149	1.309	7.81E-10
IL1B	1.187	1.106	1.275	2.31E-06	1.132	1.072	1.195	7.12E-06

### 3.3 Construction of a prognostic signature in the TCGA cohort

**FIGURE 4:**
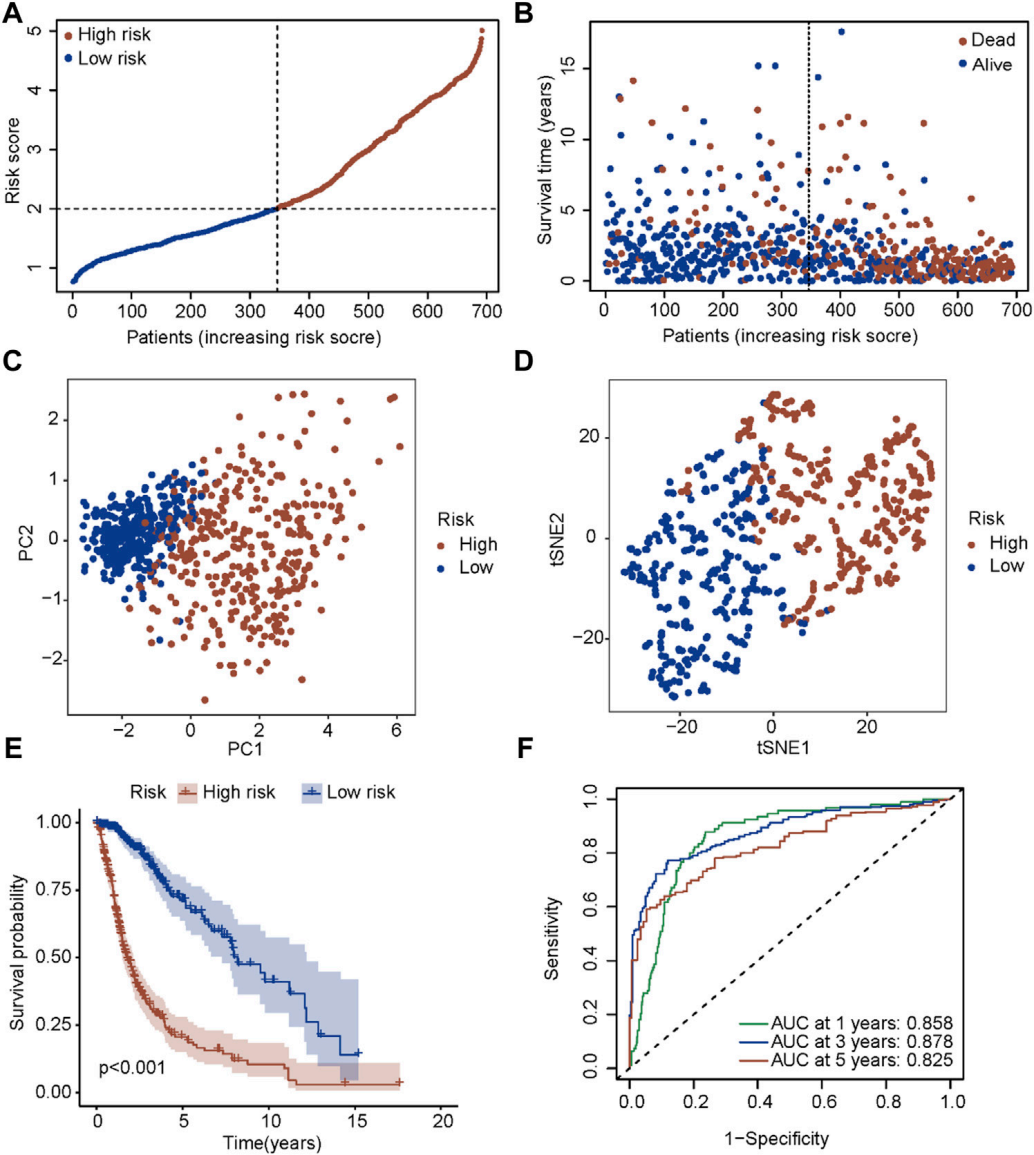
Prognostic analysis of the five-gene signature in the TCGA cohort. **(A)** The distribution and median value of the risk scores in the TCGA cohort. **(B)** The distributions of OS status, OS time and risk score in the TCGA cohort. **(C)** PCA plot of the TCGA cohort. **(D)** t-SNE analysis of the TCGA cohort. **(E)** Kaplan-Meier curves for the OS of patients in the high-risk group and low-risk group in the TCGA cohort. **(F)** AUC of time-dependent ROC curves verified the prognostic performance of the risk score in the TCGA cohort.

A new prognostic signature was established based on the genes corresponding to the top 10 hub nodes mentioned above. The five-gene signature (including *CASP4*, *CASP5*, *CASP8*, *GSDMD*, and *NLRC4*) was determined by the optimal value of the regularization parameter λ (Figures 3C, D), which divided patients into high-risk or low-risk groups based on the median cut-off value (Figure 4A). The formula for calculating the risk score is as follows: Risk score = (0.184 × GSDMD expression level) + (0.784 × CASP4 expression level) + [(−0.085) × CASP5 expression level) + (0.109) × CASP8 expression level) + ((−0.140) × NLRC4 expression level]. Figure 4B showed that patients in the high-risk group had a higher mortality rate and shorter survival time compared with patients in the low-risk group. Reduced dimension visualization of PCA and t-SNE arrived at similar conclusions that patients from different risk groups distributed in two directions (Figures 4C, D). As shown in Figure 4E, the OS of patients in high-risk group is markedly shorter than patients in the low-risk group (*p* < 0.001). The time-dependent ROC showed that the area under the curve (AUC) reached 0.858 at 1 year, 0.878 at 3 years, and 0.825 at 5 years (Figure 4F), which demonstrated excellent prognostic performance of this signature.

### 3.4 Validation of the prognostic signature in the CGGA cohort

**FIGURE 5:**
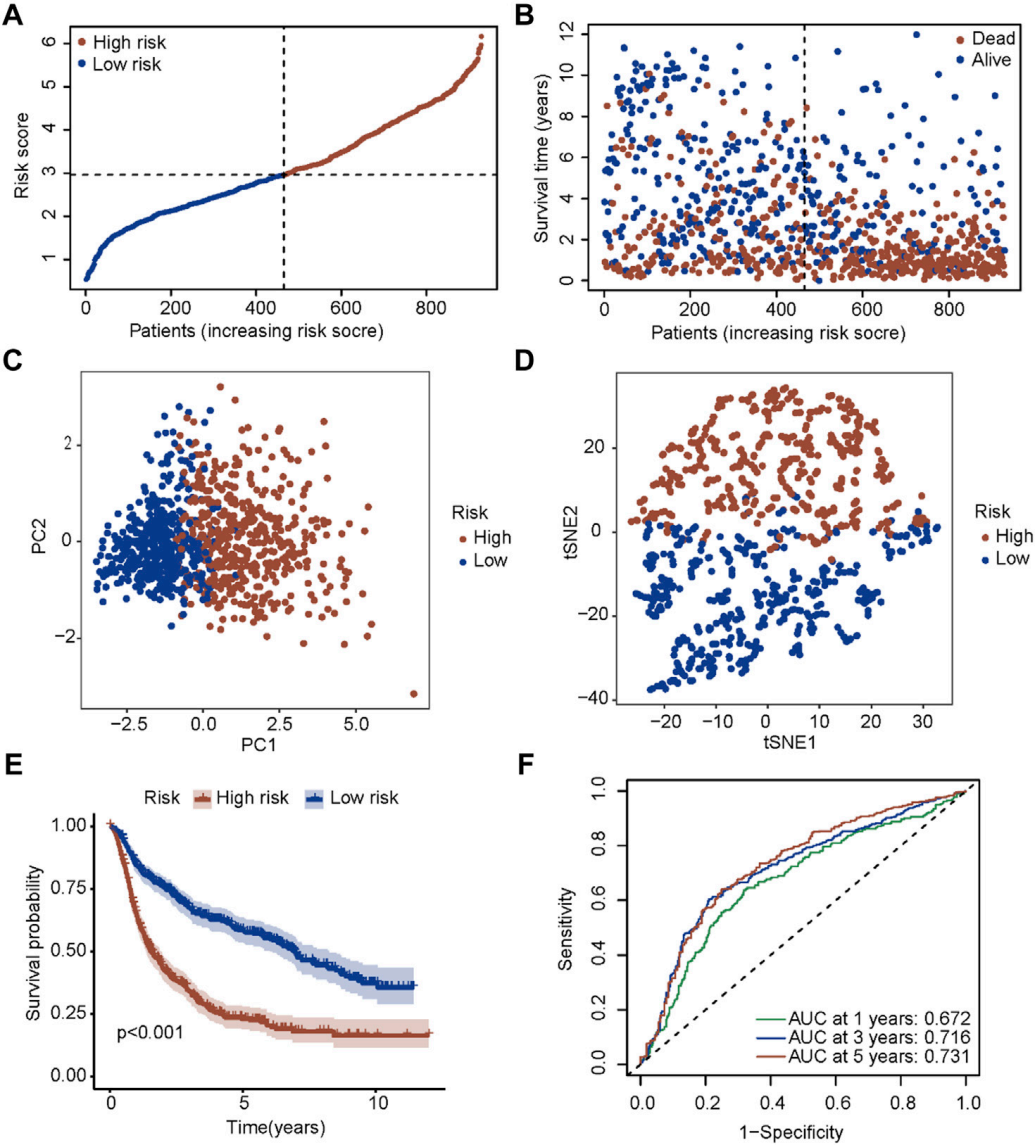
Validation of the five-gene signature in the CGGA cohort. **(A)** The distribution and median value of the risk scores in the CGGA cohort. **(B)** The distributions of OS status, OS time and risk score in the CGGA cohort. **(C)** PCA plot of the CGGA cohort. **(D)** t-SNE analysis of the CGGA cohort. **(E)** Kaplan-Meier curves for the OS of patients in the high-risk group and low-risk group in the CGGA cohort. **(F)** AUC of time-dependent ROC curves in the CGGA cohort.

To test the robustness of the signature established in the TCGA cohort, patients in the CGGA cohort were also divided into two risk groups according to the median value calculated by the same formula (Figure 5A). Similar to the results obtained from the TCGA cohort, patients with a higher risk score had a shorter survival time (Figure 5B). Also, both PCA and t-SNE analyses showed that patients in different risk groups distributed in discrete directions (Figures 5C, D). Subsequently, patients in the high-risk group showed a markedly worse survival compared with patients in the low-risk group (*p* < 0.001, Figure 5E). Moreover, the AUC reached 0.672 at 1 year, 0.716 at 3 years, and 0.731 at 5 years (Figure 5F), validating the robustness of this model.

### 3.5 Independent prognostic value of the PRGs signature

**FIGURE 6:**
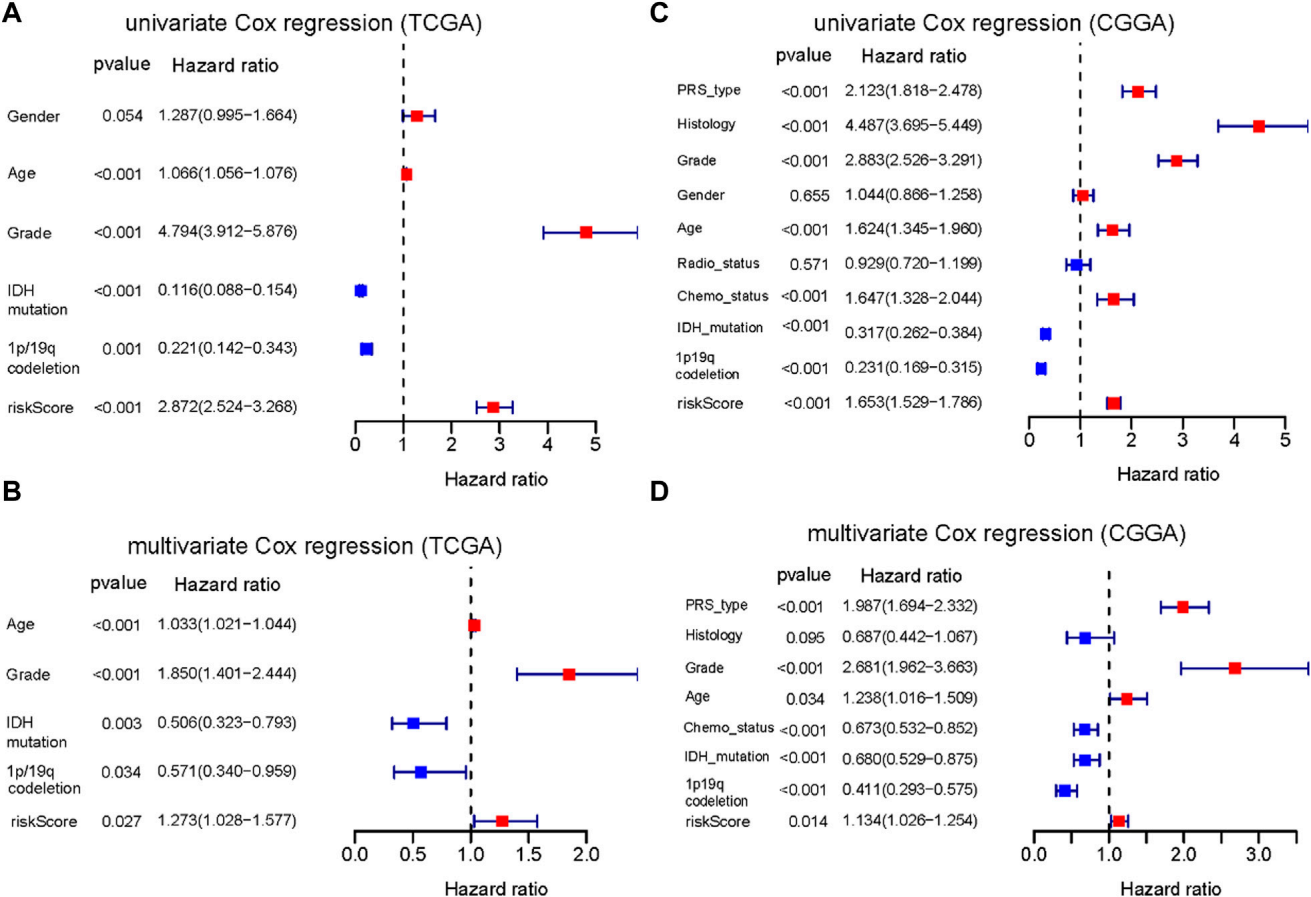
Results of the univariate and multivariate Cox regression analyses regarding OS. **(A)** Result of the univariate Cox regression analyses in the TCGA training cohort. **(B)** Result of the multivariate Cox regression analyses in the TCGA training cohort. **(C)** Result of the univariate Cox regression analyses in the CGGA training cohort. **(D)** Result of the multivariate Cox regression analyses in the CGGA training cohort.

Univariate and multivariate Cox regression analyses were performed to identify whether the risk score is an independent prognostic predictor of OS. In univariate Cox regression analysis, the risk score was obviously related to OS in both TCGA and CGGA cohort (TCGA cohort: HR = 2.872, 95% CI = 2.524–3.268, *p* < 0.001; CGGA cohort: HR = 1.653, 95% CI = 1.529–1.786, *p* < 0.001) (Figures 6A, C). After adjustment for confounding factors, the risk score was still shown to be an independent predictor of OS in the multivariate Cox regression analysis (TCGA cohort: HR = 1.273, 95% CI = 1.028–1.577, *p* = 0.027; CGGA cohort: HR = 1.134, 95% CI = 1.026–1.254, *p* = 0.014) (Figures 6B, D).

**FIGURE 7:**
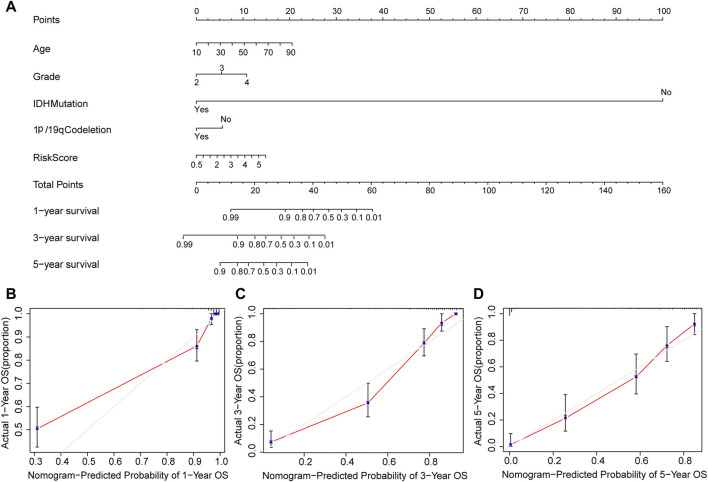
Construction of a nomogram. **(A)** Validation of the nomogram in the TCGA cohort. **(B)** Calibration plot used to predict the 1-year survival. **(C)** Calibration plot used to predict the 3-year survival. **(D)** Calibration plot used to predict the 5-year survival.

Furthermore, we constructed a nomogram to predict 1-year, 3-year, and 5-year OS of glioma patients using five prognostic factors including age, grade, *IDH*, 1p/19q and risk score (Figure 7A). The results of the calibration curve shown in Figures 7B–D showed that the survival rate obtained by the model is consistent with the actual survival rate.

### 3.6 Profiles of signature genes in glioma

**FIGURE 8:**
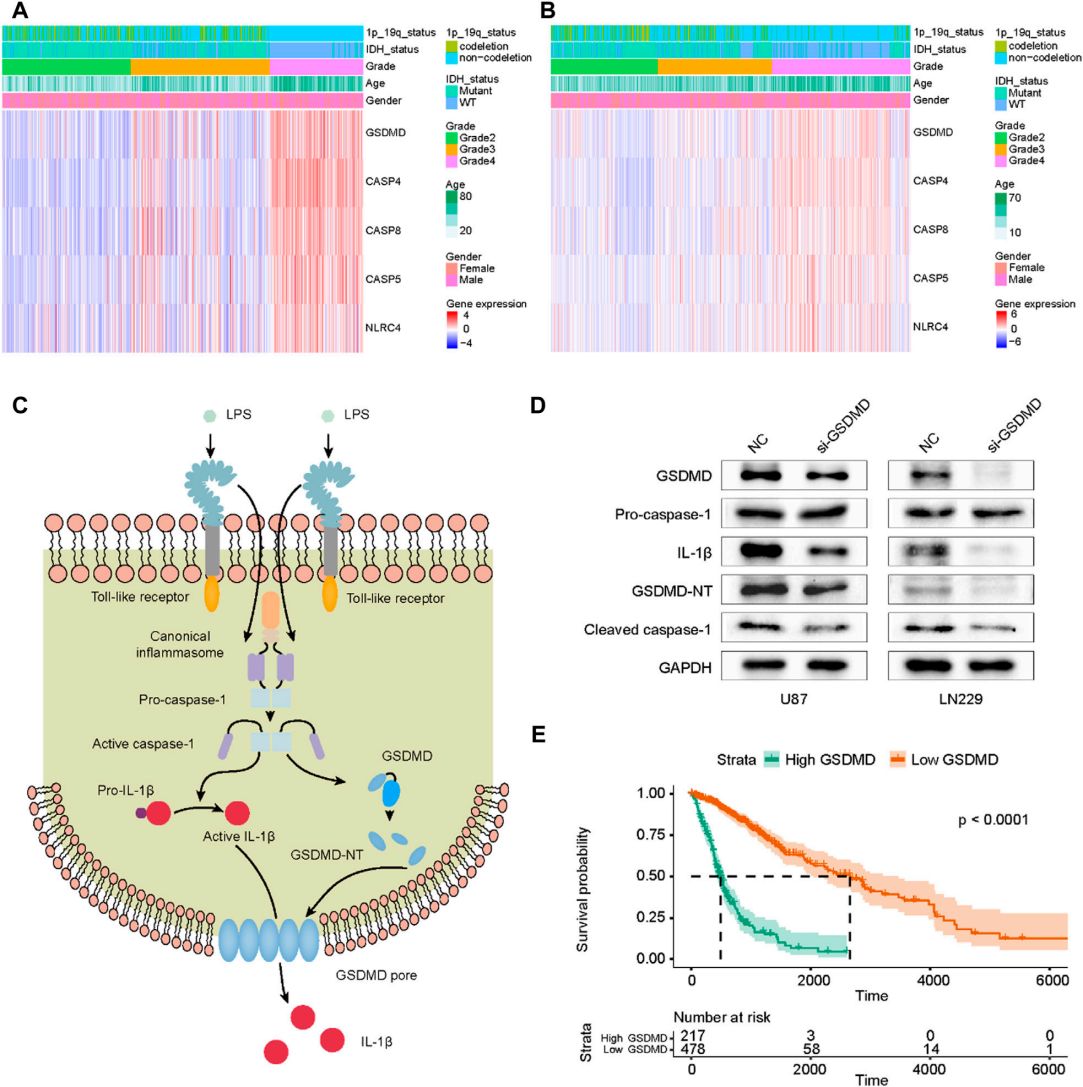
Profile of signature genes expression in gliomas and functional validation of GSDMD in pyroptosis. **(A)** The heatmap of five PRGs in TCGA cohort. **(B)** The heatmap of five PRGs in CGGA cohort. **(C)** The GSDMD-dependent pyroptosis signaling pathway regulated by caspase-1. **(D)** After siRNA transfection, the expressions of GSDMD, Pro-caspase-1, IL-1β, GSDMD-NT and cleaved caspase-1 were detected by Western Blot. **(E)** Correlation of GSDMD expression level with glioma prognosis.

Pan-cancer analysis showed that these five signature genes were highly expressed in a variety of tumors including GBM and LGG (Supplementary Figure S1). We also compared the expression differences of these genes in glioma and normal tissues, and the results demonstrated that their expression in gliomas was significantly higher than that in normal tissues. The ROC curve reflected the diagnostic efficiency of these genes (Supplementary Figure S2). Then, we analyzed the relationship between the expression level of each signature gene and the clinicopathological features of glioma patients. These results showed that these five signature genes were highly expressed in high-grade glioma, IDH wild-type, 1p/19q non-codeletion subtype and elderly patients (Figures 8A, B; Supplementary Figure S3).

GSDMD, as a key effector molecule of cell pyroptosis, is activated to mediate the formation of cell membrane pores, ultimately causing cytokine release and inflammatory cell death (Hu et al., 2020). Figure 8C showed the GSDMD-dependent pyroptosis signaling pathway. After GSDMD knockdown in glioma cells, the expression of IL-1β, cleaved caspase-1 and GSDMD-NT decreased, and the expression level of pro-caspase-1 was not affected (Figure 8D). We also evaluated the survival of glioma patients using TCGA data and found that the expression level of GSDMD was significantly associated with poor prognosis (Figure 8E). These results indicated that GSDMD was involved in mediating pyroptosis in gliomas and its expression level had a prognostic value in glioma patients.

### 3.7 Immune infiltration status in the TCGA and the CGGA cohort

**FIGURE 9:**
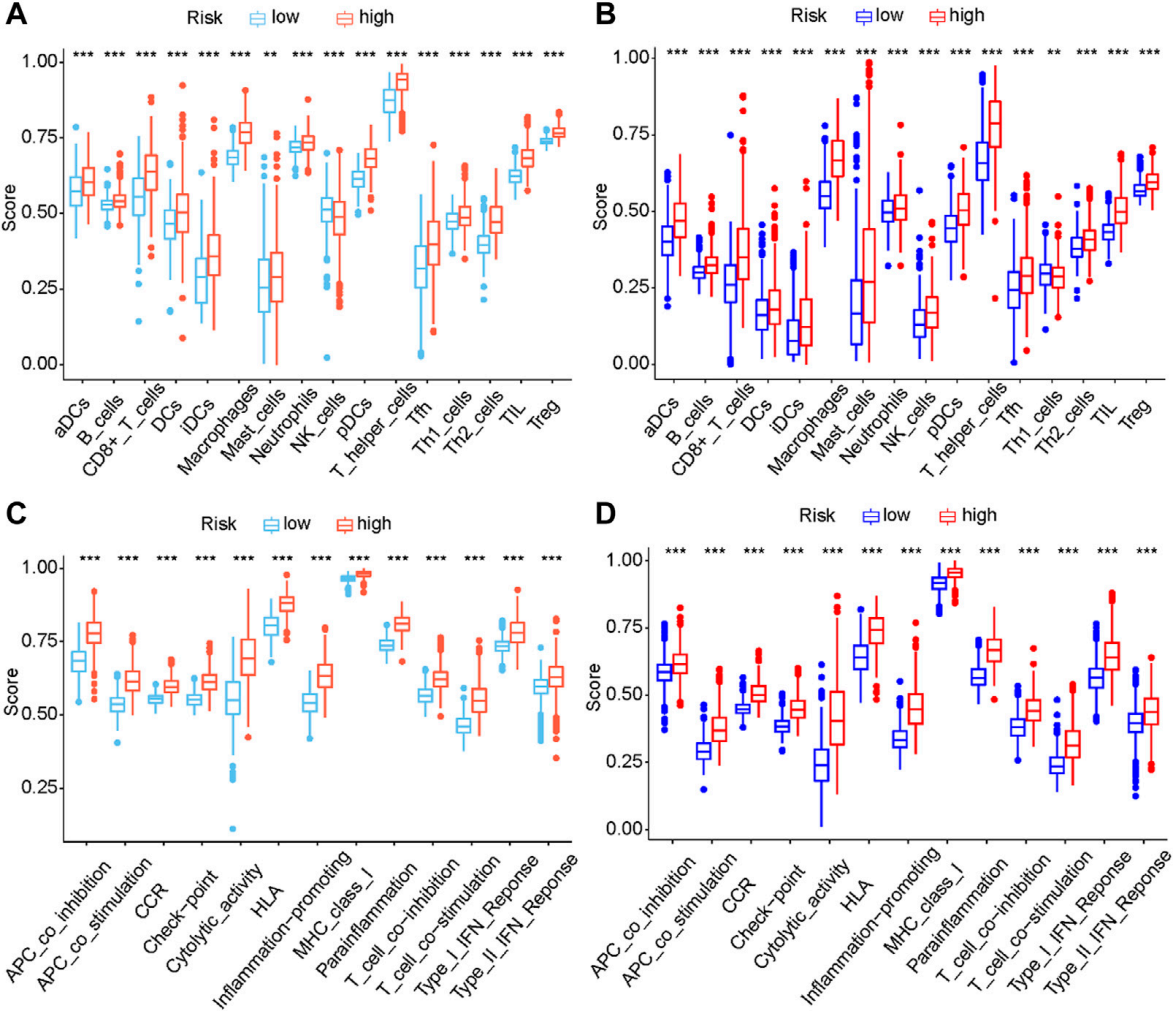
Comparison of the ssGSEA scores between different risk groups in the TCGA cohort **(A, C)** and CGGA cohort **(B, D)** The scores of sixteen immune cell types **(A, B)** and thirteen immune-related functions **(C, D)** were displayed in boxplots. (aDC, Activated dendritic cell; iDC, Immature dendritic cell; pDC, Plasmacytoid dendritic cell; Tfh, T follicular helper cell; Th2, T helper 2; TIL, Tumor infiltrating lymphocyte; Treg, Regulatery T Cell; HLA, Human leukocyte antigen; APC, Antigen presenting cell; CCR, Cytokine-cytokine receptor; Adjusted *p* values were showed as: ns, not significant; ***, *p* < 0.001.).

Pyroptosis can regulate the immune response through the release of immune stimulatory factors (Li et al., 2021). To explore the immune infiltration status between two risk groups, the enrichment scores of different immune cell types and immune-related functions were quantified by ssGSEA. In the TCGA cohort, compared with the low-risk group, the high-risk group had a higher scores of immune cells including CD8^+^ T Cells, iDCs, macrophages, pDCs, Th2 cells, TIL and Treg, etc (Figure 9A). Notably, the scores of macrophages between two different risk groups have the most significant difference. Additionally, the scores of APC co-stimulation, CCR, checkpoint molecules, type I IFN response and type II IFN response of the high-risk group were evidently higher than those of the low-risk group (*p* < 0.001, Figure 9C). The differences of immune infiltration status between two different risk groups have been verified in CGGA (*p* < 0.001, Figures 9B, D). Additionally, we performed ssGSEA to analyze the correlation between the expressions of five signature genes and immune infiltrating cells. The results of ssGSEA indicated that these five signature genes were closely associated with a variety of immune infiltrating cells (Supplementary Figure S4).

## 4 Discussion

Cell death is a fundamental physiological process, which includes the three most widely known patterns, namely, apoptosis, necroptosis, and pyroptosis (Man et al., 2017). Pyroptosis, a new type of programmed and inflammatory death found after apoptosis and necroptosis, is involved in the occurrence and progression of multiple tumors (Xia et al., 2019). With the development of research, the relationship between pyroptosis and tumors is increasingly understood and provides inspirations for treatment strategy. Cancer cells, including glioma, have evolved multiple mechanisms to evade programmed cell death in order to maintain their survival (Fulda, 2018). However, the current research on the biological value and roles of pyroptosis in the occurrence and progression of glioma is limited.

Pyroptosis is a gasdermins-mediated programmed cell death induced by caspase-1/4/5/11 and has been extensively studied in multiple diseases (Maltez et al., 2015). The GSDM protein family, especially GSDMD and GSDME, are important mediators of pyroptosis (Hsu et al., 2021). In the process of pyroptosis, GSDM protein can be cleaved to release the GSDM-N domain, which can form holes in the cell membrane, causing cytoplasm swelling, membrane rupture, and the release of inflammatory factors into the extracellular environment (Sarhan et al., 2018). Increased inflammation creates a local environment that is conducive to tumorigenesis, and plenty of evidence have indicated that chronic inflammation plays a crucial role in carcinogenesis and tumor progression (Dinarello, 2010; Carmi et al., 2013; Zha et al., 2020). Previously, it is reported that decitabine can enhance caspase-3 cleavage to GSDME and cause cancer cell pyroptosis, indicating that targeting pyroptosis might be a promising therapeutic strategy for cancer (Zhao et al., 2020). Furthermore, another research suggests that kaempferol inhibits glioma cell proliferation and tumor growth by inducing pyroptosis (Chen et al., 2020). Specifically, kaempferol can induce autophagy by increasing reactive oxygen species, and ultimately trigger the pyroptosis of glioma cells. Therefore, it is reasonable to believe the perspective applications of pyroptosis in glioma therapies.

In the present work, one hundred and eighteen genes involved in pyroptosis-related gene sets were obtained from MSigDB and GeneCards. We performed consensus clustering analysis and divided glioma patients into two clusters. Consequently, the OS of cluster 2 was significantly better than that of cluster 1, indicating the prognostic value of PRGs in gliomas. Next, we constructed protein-protein interaction (PPI) network for the screened PRGs and identified the top 10 hub nodes using the Cytoscape’s Cytohubba plug-in. Then, we constructed a novel five-gene signature for prognosis prediction of patients with glioma. The risk score of this signature was an independent prognostic factor of glioma and patients in the high-risk group had a worse survival. Calibration plots demonstrated that the nomogram combining the risk score with conventional clinical prognostic factors performed well in predicting survival for glioma patients. A previous study reported a comprehensive analysis for the role of pyroptosis in glioma, which constructed a prognostic signature based on fifteen pyroptosis-related genes and analyzed the molecular classification and immunity of glioma (Chen et al., 2021). The three genes CASP4, CASP5, and CASP8 contained in our gene signature are consistent with the fifteen-gene signature in the previous study. These two gene signatures validated each other, reflecting the plausibility of this PRGs-based prognostic approach. Currently, there are several methods to predict glioma prognosis, including clinicopathological classification, MRI imaging, and molecular markers (such as IDH mutation status, 1p/19q codeletion, MGMT promoter methylation status, etc.) detection (Sledzinska et al., 2021). IDH mutations are positive prognostic markers in gliomas and have significant prognostic value. Additionally, MGMT promoter methylation has important significance in predicting the response to temozolomide (TMZ) in glioma patients, which indirectly reflects the prognosis of patients. Compared to the above methods, our study focused on the role of pyroptosis in the prognosis of gliomas and constructed a new predictive signature based on PRGs. This prognostic signature was combined with clinical characteristics and provided a more comprehensive and individualized approach to prognostic assessment. Although the prognostic signature requires more prospective data to further test its clinical utility, our results suggest that the prognostic signature based on five genes may be a powerful indicator of glioma patient survival.

The novel prognostic signature established in our study contained five PRGs. GSDMD, a member of the gasdermins protein family, is considered to be the executioner of pyroptosis (Broz et al., 2020). The protein contains an inhibitory C-terminal domain and a pore-forming N-terminal domain (GSDMD-NT). GSDMD can be cleaved by caspase-1/4/5/11 to expose the N-terminal domain, casuing the formation of membrane pores (Kayagaki et al., 2015; Shi et al., 2015). Caspases are a family of cysteine proteases, which play a key role in the process of inflammation, cell death and disease (Van Opdenbosch and Lamkanfi, 2019). CASP4, CASP5, and CASP8 encode the proteins caspase-4, caspase-5, and caspase-8, respectively. Caspase-4 and caspase-5 activated by lipopolysaccharide promote GSDMD-mediated pyroptosis, while caspase-8 is mainly involved in triggering death receptor-mediated apoptosis (Downs et al., 2020; Mandal et al., 2020). In glioblastoma, caspase-8 promotes the expression of various cytokines, angiogenesis, and tumorigenesis (Fianco et al., 2018). However, the roles of *CASP4* and *CASP5* in gliomas has not been reported. NLRC4 is an apoptosis-related protein that interacts with caspase-1 and induces the activation of inflammasomes (Duncan and Canna, 2018). As a member of the NOD-like receptor family, NLRC4 is recognized by NAIP subfamily proteins and binds to form NAIP-NLRC4 inflammasome (Kay et al., 2020). Jaehoon Lim et al. has previously reported that upregulation of NLRC4 inflammasome is associated with poor prognosis in glioma patients (Lim et al., 2019). In this study, we analyzed the relationship between the expression of these five PRGs and the prognosis and clinicopathological characteristics of glioma patients. The results suggested that the high expression of these genes is associated with poor clinical phenotype and prognosis. Then, we selected GSDMD from these five PRGs for knockdown and found that GSDMD participated in mediating the process of pyroptosis in gliomas. Since there are few reports about PRGs in gliomas, the specific mechanisms of these genes in gliomas require further research.

Recently, pyroptosis has attracted increasing interest due to its role in activating the immune system. Research on the characteristics of pyroptosis and its roles in pathophysiological conditions has advanced our understanding of inflammation, immune responses, and tumor development (Tan et al., 2021). On the one hand, the release of cytokines produced by pyroptosis changes the immune microenvironment and promotes tumor development through immune evasion. On the other hand, the cytokines produced by pyroptosis can also recruit immune cells and activate the immune system to enhance the effect of tumor immunotherapy (Li et al., 2021). In the present study, we performed ssGSEA to explore the immune infiltration status between two different risk groups. Particularly, patients in high-risk group showed a higher proportion of immune infiltrating cells, including iDCs, pDCs, Th2 cells, TIL, Treg and macrophages. Moreover, the expression levels of these five signature genes were closely related to immune infiltrating cells. A reasonable argument is that pyroptosis of cancer cells activates anti-tumor immunity, which significantly increases the number of immune cells (Wang et al., 2020b). Nevertheless, the underlying mechanism between PRGs and immune status in glioma is still unclear, and further research is needed. Besides, previous studies have suggested that tumor-associated macrophages are involved in poor prognosis of glioma because of their roles in immune suppression and invasion (Peng et al., 2020). Therefore, increased macrophage infiltration of patients in high-risk group may be one of the reasons for their poor prognosis.

## 5 Conclusion

Our study constructed a novel PRGs signature, which could serve as a powerful tool to predict the prognosis of glioma patients. This signature could be applied to risk stratification of patients with glioma. In addition, the signature was connected with immune infiltration status, providing an improved understanding of immune response in glioma. Our results indicated that PRGs may be promising therapeutic targets of gliomas.

## Data Availability

The original contributions presented in the study are included in the article/supplementary material, further inquiries can be directed to the corresponding authors.

## Abbreviations

AUC, Area under the curve; CDF, Cumulative-distribution function; CGGA, Chinese Glioma Genome Atlas; CI, Confidence interval; CNS, Central nervous system; DEGs, Differentially expressed genes; FDR, False discovery rate; GBM, Glioblastoma; GSDM, Gasdermin; LASSO, Least absolute shrinkage and selection operator; LGG, Lower-grade gliomas; OS, Overall survival; PCA, Principal component analysis; PRGs, pyroptosis-related genes; ROC, Receiver operating characteristic; ssGSEA, Single-sample gene set enrichment analysis; TCGA, The Cancer Genome Atlas; t-SNE, t-distributed stochastic neighbor embedding.
