# Supplementary material for: Development and validation of a pyroptosis-related genes signature for risk stratification in gliomas
Source: Front Genet. 2023 Feb 13;14:1087563. doi: 10.3389/fgene.2023.1087563 (PMC9968976; doi:10.3389/fgene.2023.1087563)
Supplement: Supplementary file 1 [file DataSheet1.docx]

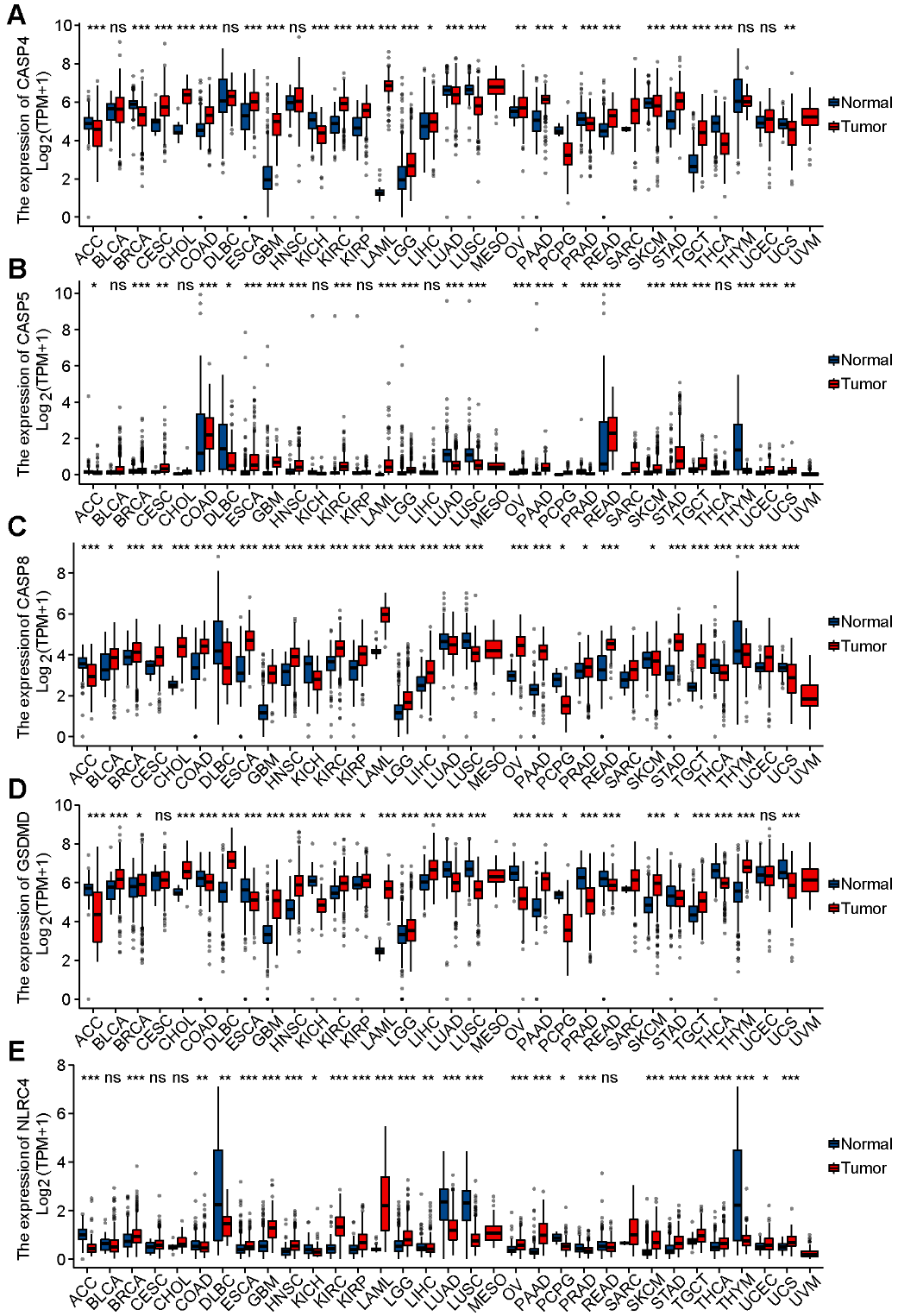


**Supplementary Figure 1. Pan-cancer analysis of five signature genes. The differential expression of each signature gene（*CASP4*, *CASP5*, *CASP8*, *GSDMD* and *NLRC4*）between multiple tumor tissues and normal tissues.**

**
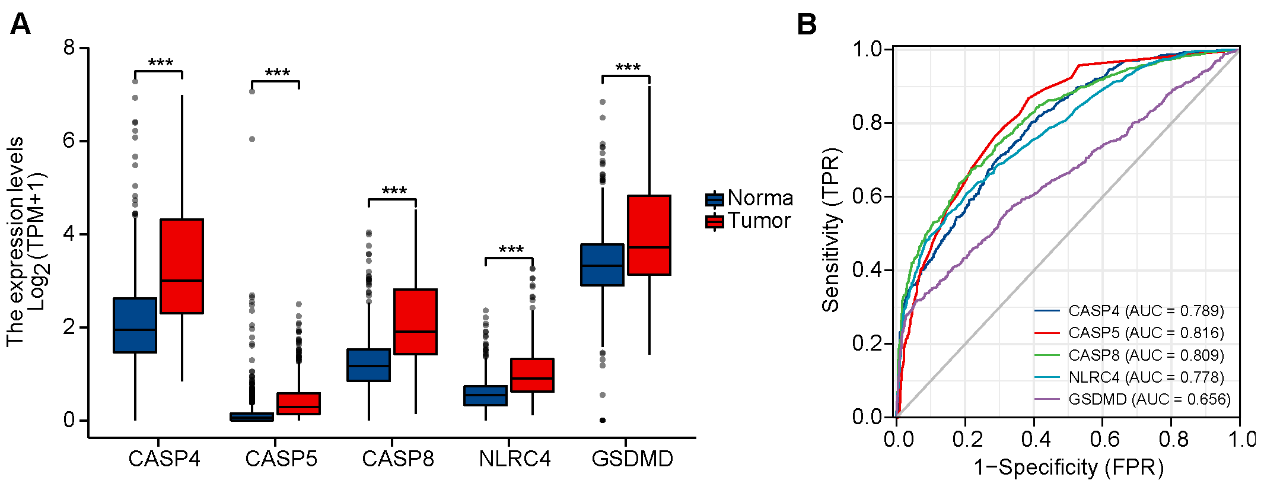
**

**Supplementary Figure 2. Differential expression analysis of five signature genes between glioma and normal tissues. (A). The expression levels of *CASP4*, *CASP5*, *CASP8*, *GSDMD* and *NLRC4* in glioma and normal tissues. (B). The ROC curve analysis of five signature genes.**

**
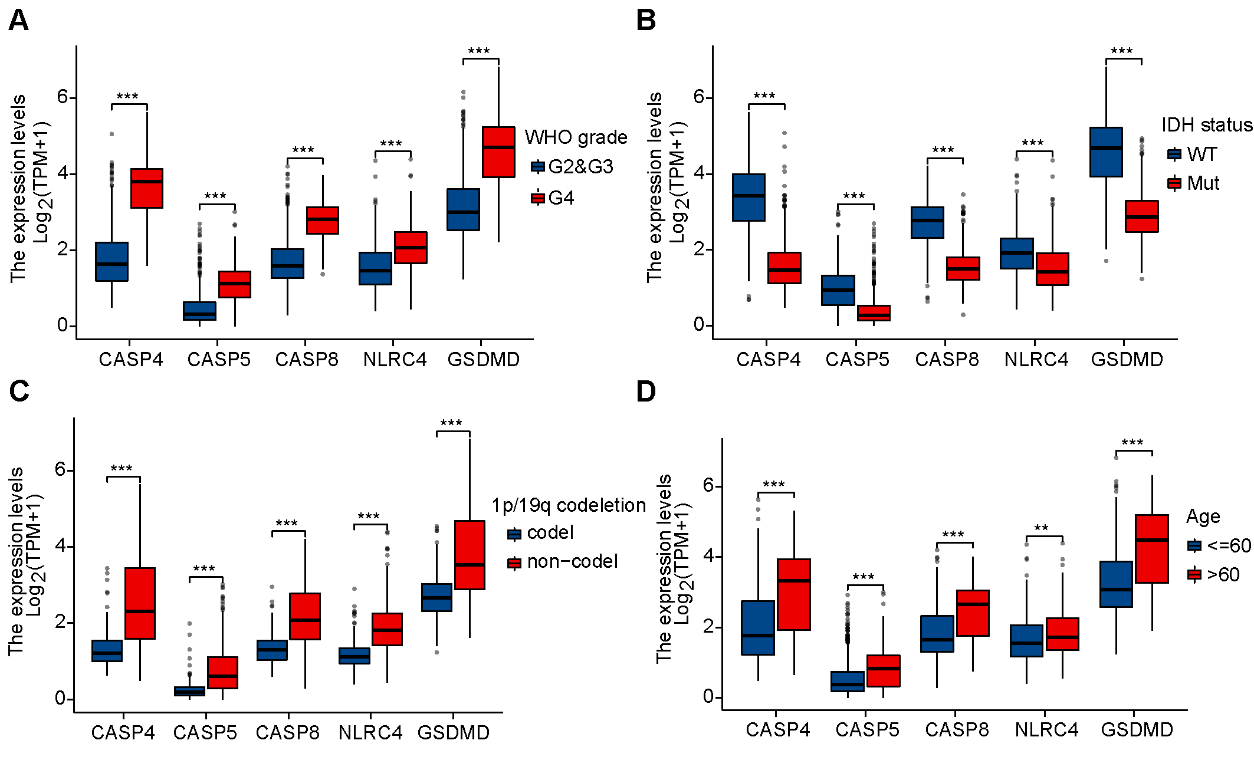
**

**Supplementary Figure 3. The relationship between the expression levels of five signature genes and the clinicopathological features (WHO grade, IDH-mutant status, 1p/19q codeletion and age).**

**
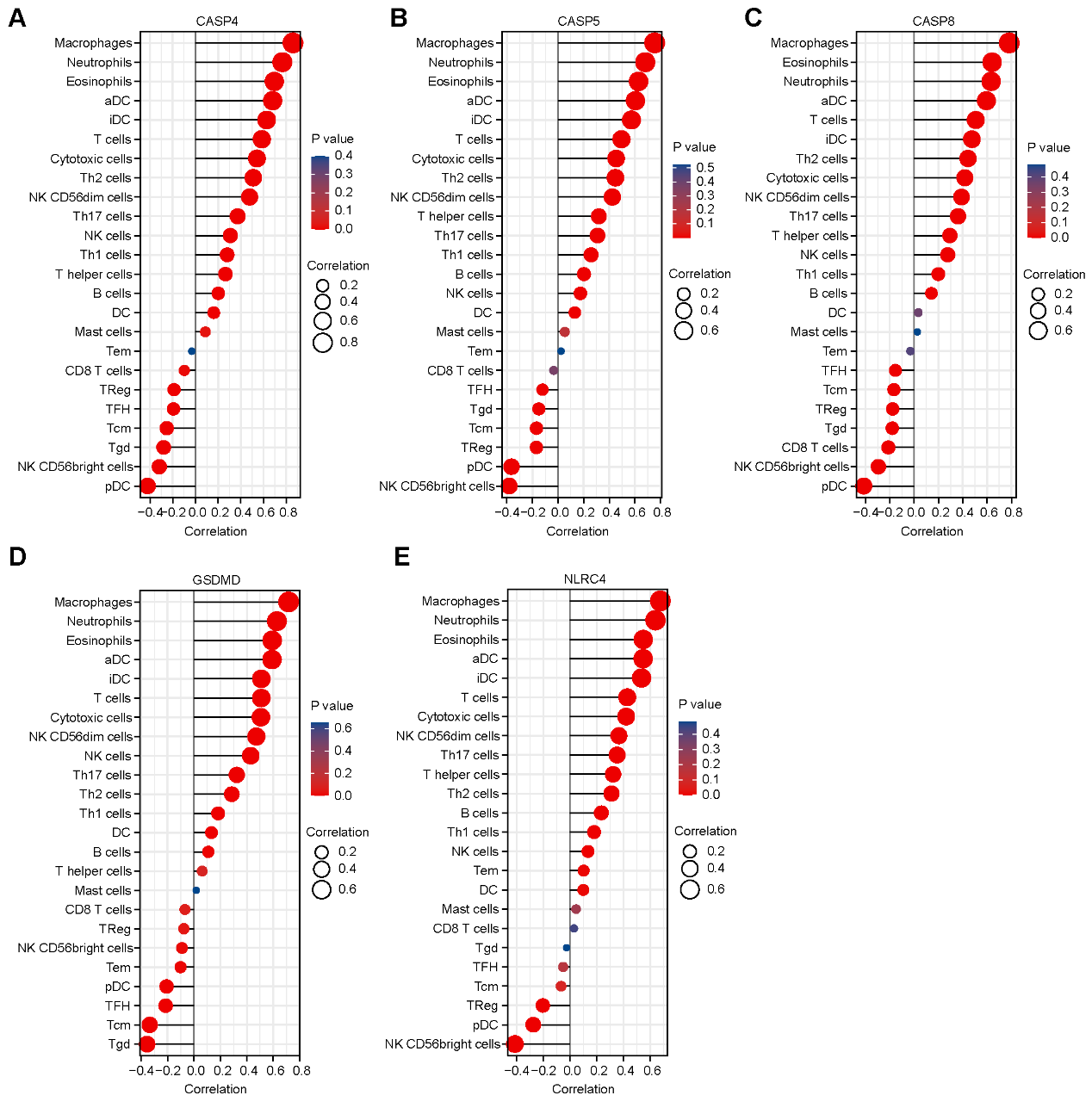
**

**Supplementary Figure 4. The correlation between the expression levels of five signature genes（*CASP4*, *CASP5*, *CASP8*, *GSDMD* and *NLRC4*）and infiltrating immune cell types in glioma.**

**
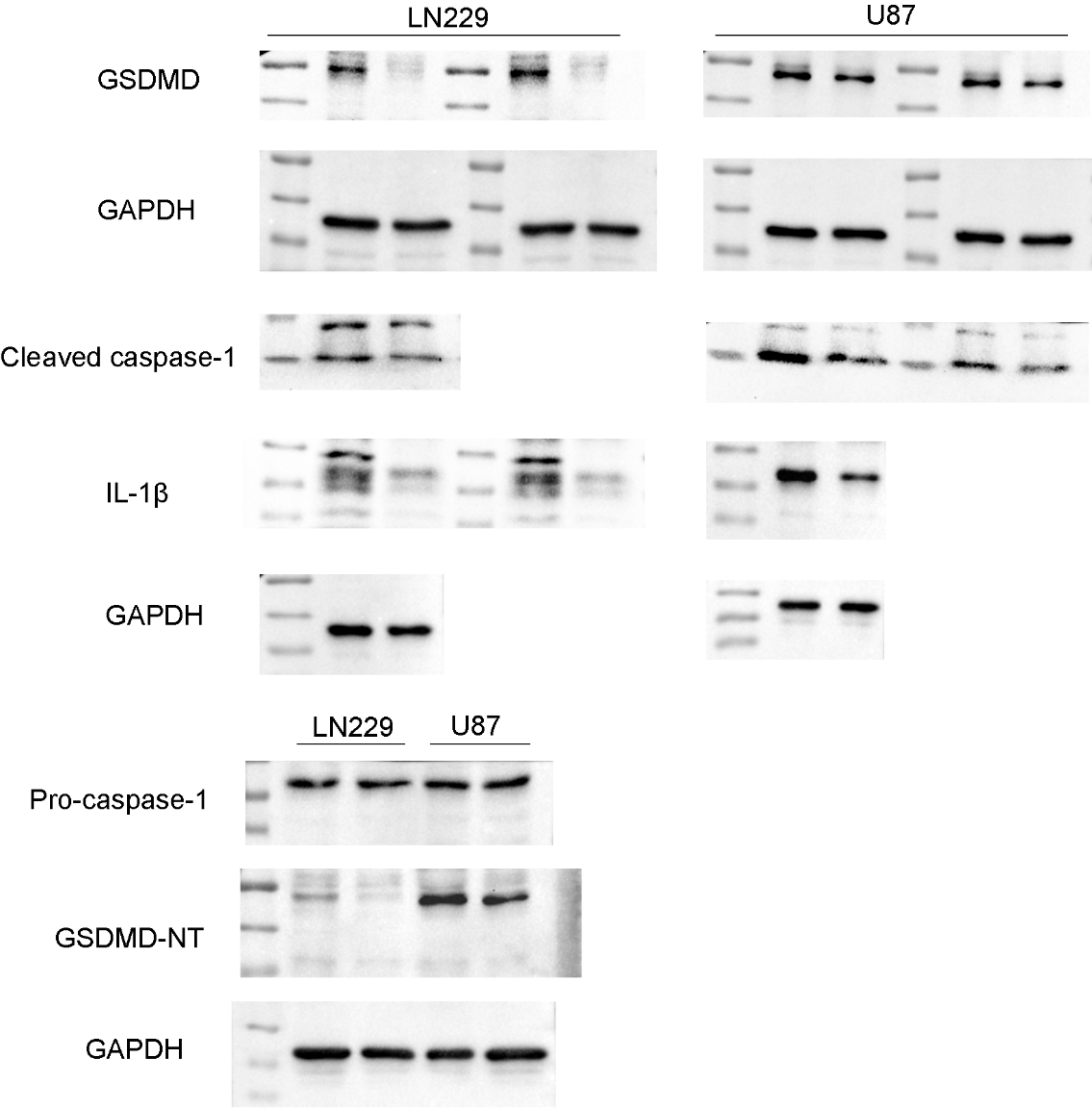
**

**Supplementary Figure 5. Original Western Blot Images.**

**Supplementary Table 1．One hundred and eighteen genes involved in pyroptosis-related gene sets**

| **GOBP_PYROPTOSIS** | **REACTOME_PYROPTOSIS** | **Genecards (Relevance Score>1)** | **Union Set** |
| --- | --- | --- | --- |
| AIM2 | BAK1 | ADORA1 | AIM2 |
| APIP | BAX | ADORA2A | APIP |
| CASP1 | CASP1 | ADORA2B | CASP1 |
| CASP4 | CASP3 | ADORA3 | CASP4 |
| CASP8 | CASP4 | AGER | CASP8 |
| DHX9 | CASP5 | AIM2 | DHX9 |
| GSDMA | CHMP2A | APIP | GSDMA |
| GSDMB | CHMP2B | APOE | GSDMB |
| GSDMC | CHMP3 | BRD4 | GSDMC |
| GSDMD | CHMP4A | BSG | GSDMD |
| GSDME | CHMP4B | CAMP | GSDME |
| GZMA | CHMP4C | CARD8 | GZMA |
| GZMB | CHMP6 | CASP1 | GZMB |
| NAIP | CHMP7 | CASP3 | NAIP |
| NLRC4 | CYCS | CASP4 | NLRC4 |
| NLRP1 | ELANE | CASP5 | NLRP1 |
| NLRP9 | GSDMD | CASP6 | NLRP9 |
| ZBP1 | GSDME | CASP8 | ZBP1 |
|  | GZMB | CD274 | BAK1 |
|  | HMGB1 | CDKN2B-AS1 | BAX |
|  | IL18 | CEBPB | CASP3 |
|  | IL1A | CPTP | CASP5 |
|  | IL1B | CRTAC1 | CHMP2A |
|  | IRF1 | CTSV | CHMP2B |
|  | IRF2 | DDX3X | CHMP3 |
|  | TP53 | DHX9 | CHMP4A |
|  | TP63 | DPP8 | CHMP4B |
|  |  | DPP9 | CHMP4C |
|  |  | EEF2K | CHMP6 |
|  |  | ELAVL1 | CHMP7 |
|  |  | FGF21 | CYCS |
|  |  | FOXO3 | ELANE |
|  |  | GBP1 | HMGB1 |
|  |  | GBP5 | IL18 |
|  |  | GJA1 | IL1A |
|  |  | GSDMA | IL1B |
|  |  | GSDMB | IRF1 |
|  |  | GSDMC | IRF2 |
|  |  | GSDMD | TP53 |
|  |  | GSDME | TP63 |
|  |  | GZMA | HNP1 |
|  |  | GZMB | NLRP3 |
|  |  | HDAC6 | CARD8 |
|  |  | HMGB1 | DPP9 |
|  |  | HNP1 | DPP8 |
|  |  | IL18 | PYCARD |
|  |  | IL1B | TREM2 |
|  |  | IL32 | FOXO3 |
|  |  | IRF3 | CASP6 |
|  |  | KCNQ1OT1 | TXNIP |
|  |  | KLF3-AS1 | DDX3X |
|  |  | MALAT1 | GBP1 |
|  |  | MALT1 | GJA1 |
|  |  | MEG3 | PRDM1 |
|  |  | METTL3 | UBR2 |
|  |  | MIR125A | CPTP |
|  |  | MIR135B | VDR |
|  |  | MIR155 | BRD4 |
|  |  | MIR204 | NEK7 |
|  |  | MIR21 | CRTAC1 |
|  |  | MIR214 | NFE2L2 |
|  |  | MIR22 | AGER |
|  |  | MIR223 | TET2 |
|  |  | MIR25 | UTS2 |
|  |  | MIR30C1 | CTSV |
|  |  | MIR485 | NFKB1 |
|  |  | MIR497 | APOE |
|  |  | MIR9-1 | SDHB |
|  |  | MIR9-2 | EEF2K |
|  |  | MIR9-3 | P2RX7 |
|  |  | MRE11 | CD274 |
|  |  | MST1 | FGF21 |
|  |  | NAIP | CEBPB |
|  |  | NEK7 | TFAM |
|  |  | NFE2L2 | BSG |
|  |  | NFKB1 | IL32 |
|  |  | NLRC4 | MALT1 |
|  |  | NLRP1 | STK4 |
|  |  | NLRP3 | MST1 |
|  |  | NLRP9 | PRF1 |
|  |  | P2RX7 | ELAVL1 |
|  |  | PARP1 | HDAC6 |
|  |  | PECAM1 | SQSTM1 |
|  |  | PRDM1 | IRF3 |
|  |  | PRF1 | ZDHHC1 |
|  |  | PTEN | STING1 |
|  |  | PYCARD | PTEN |
|  |  | SDHB | ADORA1 |
|  |  | SQSTM1 | ADORA2B |
|  |  | STING1 | ADORA3 |
|  |  | STK4 | ADORA2A |
|  |  | TET2 | METTL3 |
|  |  | TFAM | PECAM1 |
|  |  | TP53 | TRIM31 |
|  |  | TREM2 | CAMP |
|  |  | TRIM31 | MRE11 |
|  |  | TXNIP | PARP1 |
|  |  | UBR2 | GBP5 |
|  |  | UTS2 | KCNQ1OT1 |
|  |  | VDR | MIR223 |
|  |  | ZBP1 | MALAT1 |
|  |  | ZDHHC1 | MIR22 |
|  |  |  | MIR125A |
|  |  |  | MIR30C1 |
|  |  |  | MIR214 |
|  |  |  | MIR155 |
|  |  |  | KLF3-AS1 |
|  |  |  | MEG3 |
|  |  |  | MIR21 |
|  |  |  | MIR135B |
|  |  |  | MIR485 |
|  |  |  | CDKN2B-AS1 |
|  |  |  | MIR204 |
|  |  |  | MIR9-1 |
|  |  |  | MIR9-3 |
|  |  |  | MIR9-2 |
|  |  |  | MIR497 |
|  |  |  | MIR25 |

**Supplementary** **Table 2．The clinical characteristics of two clusters.**

| **Characteristic** | **Cluster 1** | **Cluster 2** | **p** |
| --- | --- | --- | --- |
| **Total number, N** | 455 | 170 |  |
| **WHO grade, n (%)** |  |  | < 0.001 |
| Grade2 | 133 (21.3%) | 89 (14.2%) |  |
| Grade3 | 175 (28%) | 67 (10.7%) |  |
| Grade4 | 147 (23.5%) | 14 (2.2%) |  |
| **IDH status, n (%)** |  |  | < 0.001 |
| Mutant | 239 (38.2%) | 143 (22.9%) |  |
| Wild-type | 216 (34.6%) | 27 (4.3%) |  |
| **1p/19q, n (%)** |  |  | 0.421 |
| Codeletion | 115 (18.4%) | 37 (5.9%) |  |
| Non-codeletion | 340 (54.4%) | 133 (21.3%) |  |
| **Gender, n (%)** |  |  | 0.184 |
| Female | 200 (32%) | 64 (10.2%) |  |
| Male | 255 (40.8%) | 106 (17%) |  |
| **Age, n (%)** |  |  | < 0.001 |
| ≤ 60 | 333 (53.3%) | 154 (24.6%) |  |
| > 60 | 122 (19.5%) | 16 (2.6%) |  |
